# Supplementary material for: Comparative Analysis of Water Stress Regimes in Avocado Plants during the Early Development Stage
Source: Plants (Basel). 2024 Sep 23;13(18):2660. doi: 10.3390/plants13182660 (PMC11435208; doi:10.3390/plants13182660)
Supplement: Supplementary file 1 [file plants-13-02660-s001.zip › plants-3196542-supplementary.pdf]

Table S1. Chemical and physical characteristics of soil substrate used in avocado nursery

**Characteristic**	**Value**
Texture	Sandy-loam
pH	5.5
Electrical conductivity (EC)	0.34 dS m^{-1}
Soil Organic Matter (SOM)	18%
Total Phosphorus (P_{Total})	7 mg kg^{-1}
Soluble Phosphorus ($P_{Soluble}$)	0.013 mg L^{-1}
Sulfur (S)	26 mg kg^{-1}
Calcium (Ca)	3.4 $cmol^{(+)}$ kg^{-1}
Magnesium (Mg)	1.3 $cmol^{(+)}$ kg^{-1}
Potassium (K)	0.69 $cmol^{(+)}$ kg^{-1}
Cation Exchange Capacity (CEC)	5.33 $cmol^{(+)}kg^{-1}$
Ca_{Sat}	62.9%
Mg_{Sat}	24.2%
K_{Sat}	12.9%
Iron (Fe)	53 mg kg^{-1}
Manganese (Mn)	7 mg kg^{-1}
Copper (Cu)	3 mg kg^{-1}
Zinc (Zn)	2 mg kg^{-1}
Boron (B)	0.09 mg kg^{-1}
Ca/Mg	2.6
Mg/K	1.9
Ca/K	4.9
Ca+Mg/K	6.7
